# *MNB1* gene is involved in regulating the iron-deficiency stress response in *Arabidopsis thaliana*

**DOI:** 10.1186/s12870-022-03553-5

**Published:** 2022-03-28

**Authors:** Hui Song, Feng Chen, Xi Wu, Min Hu, Qingliu Geng, Min Ye, Cheng Zhang, Li Jiang, Shuqing Cao

**Affiliations:** School of Food and Biological Engineering, Hefei University of Technology, Hefei, 230009 China

**Keywords:** Fe-deficiency, MNB1, Reactive oxygen species, *Arabidopsis*

## Abstract

**Background:**

Iron (Fe) is an essential mineral element that involves in many biological processes important for most plants growth and development. Fe-deficiency induces a complex series of responses in plants, involving physiological and developmental changes, to increase Fe uptake from soil. However, the molecular mechanism involved in plant Fe-deficiency is not well understood.

**Results:**

Here, we found that the *MNB1* (mannose-binding-lectin 1) gene is involved in the regulation of Fe-deficiency stress response in *Arabidopsis thaliana*. The expression abundance of *MNB1* was inhibited by Fe-deficiency stress. Knockout of *MNB1* led to enhanced Fe accumulation and tolerance, whereas the *MNB1*-overexpressing plants were sensitive to Fe-deficiency stress. Under conditions of normal and Fe-deficiency, lower H_2_O_2_ concentrations were detected in *mnb1* mutant plants compared to wild type. On the contrary, higher H_2_O_2_ concentrations were found in *MNB1*-overexpressing plants, which was negatively correlated with malondialdehyde (MDA) levels. Furthermore, in *mnb1* mutants, the transcription level of the Fe uptake- and translocation-related genes, *FIT*, *IRT1*, *FRO2*, *ZIF*, *FRD3*, *NAS4*, *PYE* and *MYB72*, were considerably elevated during Fe-deficiency stress, resulting in enhanced Fe uptake and translocation, thereby increasing Fe accumulation.

**Conclusions:**

Together, our findings show that the *MNB1* gene negatively controls the Fe-deficiency response in *Arabidopsis* via modulating reactive oxygen species (ROS) levels and the ROS-mediated signaling pathway, thereby affecting the expression of Fe uptake- and translocation-related genes.

**Supplementary Information:**

The online version contains supplementary material available at 10.1186/s12870-022-03553-5.

## Background

Iron (Fe) is an essential micronutrient for plant metabolism, growth and development that acts as a cofactor of metalloproteins, which participated in many fundamental biological processes [1, 2]. Although Fe is a plentiful element in the environment, it is frequently found in saline-alkaline and calcareous soils as insoluble ferric hydroxides, resulting in low Fe bioavailability for plants [3]. As a result, understanding the molecular mechanisms underpinning iron absorption and trafficking is crucial for improving iron bioavailability and content in plants. To Fe acquisition from the soil, most plants have developed two major ways to absorb Fe: (1) the reduction-based strategy (Strategy I) mechanism found in all dicots and non-graminaceous monocots, and (2) the chelation-based strategy (Strategy II) mechanism found only in graminaceous plants [4–6]. The strategy I mechanism involves protons being extruded to lower the pH of the rhizosphere, allowing *FRO2* (Ferric reductase oxidase 2) to convert ferric iron compounds to more soluble Fe^2+^, and then transporting ferrous Fe from the soil into the root epidermal cell membrane via *IRT1* (iron-regulated transporter 1) [7–9]; whereas under low Fe stress, the phytosiderophores (mugineic acids) released by strategy II graminaceous plants chelate ferric Fe, and the resultant complexes are delivered into the root cells through the Yellow-stripe 1 transporters [10, 11]. In addition, plants have developed a number of regulatory mechanisms at both the transcriptional and post-transcriptional levels to maintain Fe homeostasis, owing to the crucial biological activities of Fe. Several transcription factors have been verified to regulate Fe-deficient-stress responses in *Arabidopsis*, such as *FIT, bHLH38, bHLH39, bHLH100, bHLH101, bHLH34, bHLH104, bHLH105, bHLH115, PYE, MYB10 and MYB72* [12–22]. These findings help us understanding plant responses to Fe-deficiency stress, however, a number of novel genes involved in modulating iron homeostasis still need to be identified.

Recent research has revealed that carbohydrate-binding proteins, also known as agglutinins or lectins, are found in a wide range of plant species and have an important biological function in pathogen defense responses [23]. Plant mannose binding lectins detect specific protein-carbohydrate combinations on pathogen surfaces and serve a key role in plant defense mechanisms against pathogens [24, 25]. Because of an interaction with cell wall extracellular glycans or carbohydrates, several plant lectins are vital for bacterial defense via an indirect biochemical process. According to earlier research, all known plant lectins can be classified into 12 plant lectin clusters of small and structurally similar proteins [26]. The pepper mannose-binding lectin gene *CaMBL1* was recently discovered to play a role in microbial pathogen defense, and mannose has been confirmed to bind to *CaMBL1* [24]. Mannose is thought to have a major role in plant resistance to cadmium toxicity, according to a recent study [27]. Also, we found that *MNB1*(mannose-binding-lectin 1)*,* an Arabidopsis homolog of CaMBL1, modulates Cd tolerance [28]. However, it is unknown whether *MNB1* is participated in modulating Fe-deficiency stress.

Plant development and stress responses, such as drought, salt, and nutrient deficiency, are influenced by reactive oxygen species (ROS) [29, 30]. Several mutants defective in ROS homeostasis displayed developmental defects and sterility mainly due to ROS accumulation [30, 31]. Increased levels of ROS can cause oxidative stress in plants, which leads to programmed cell death (PCD) at the tissue and organ level [32]. DPS1, for example, regulates ROS homeostasis and thus controls panicle apical degeneration and anther cuticle growth [30]. Excess Fe has previously been shown to be damaging to plants due to the formation of hydroxyl radicals (OH˙) via the Fenton reaction, the most active ROS [33, 34]. As a result, ROS generation must be tightly controlled by the antioxidant defense system in plants [35]. The role of ROS in Fe response modulation has not been thoroughly characterized up to this point. Here, we showed that the *MNB1* gene negatively modulates Fe-deficiency response. The transcription of the *MNB1* gene was inhibited by Fe-deficiency stress, and the *mnb1* mutants showed increased Fe-deficiency tolerance. In response to Fe shortage stress, the *MNB1* gene triggered reactive oxygen species (ROS)-mediated signaling by controlling ROS levels, consequently, affecting the expression of Fe-uptake and translocation related genes, which resulted in increased Fe accumulation and tolerance.

## Results

### The *mnb1* mutants showed enhanced Fe-deficiency stress tolerance

**Fig. 1:**
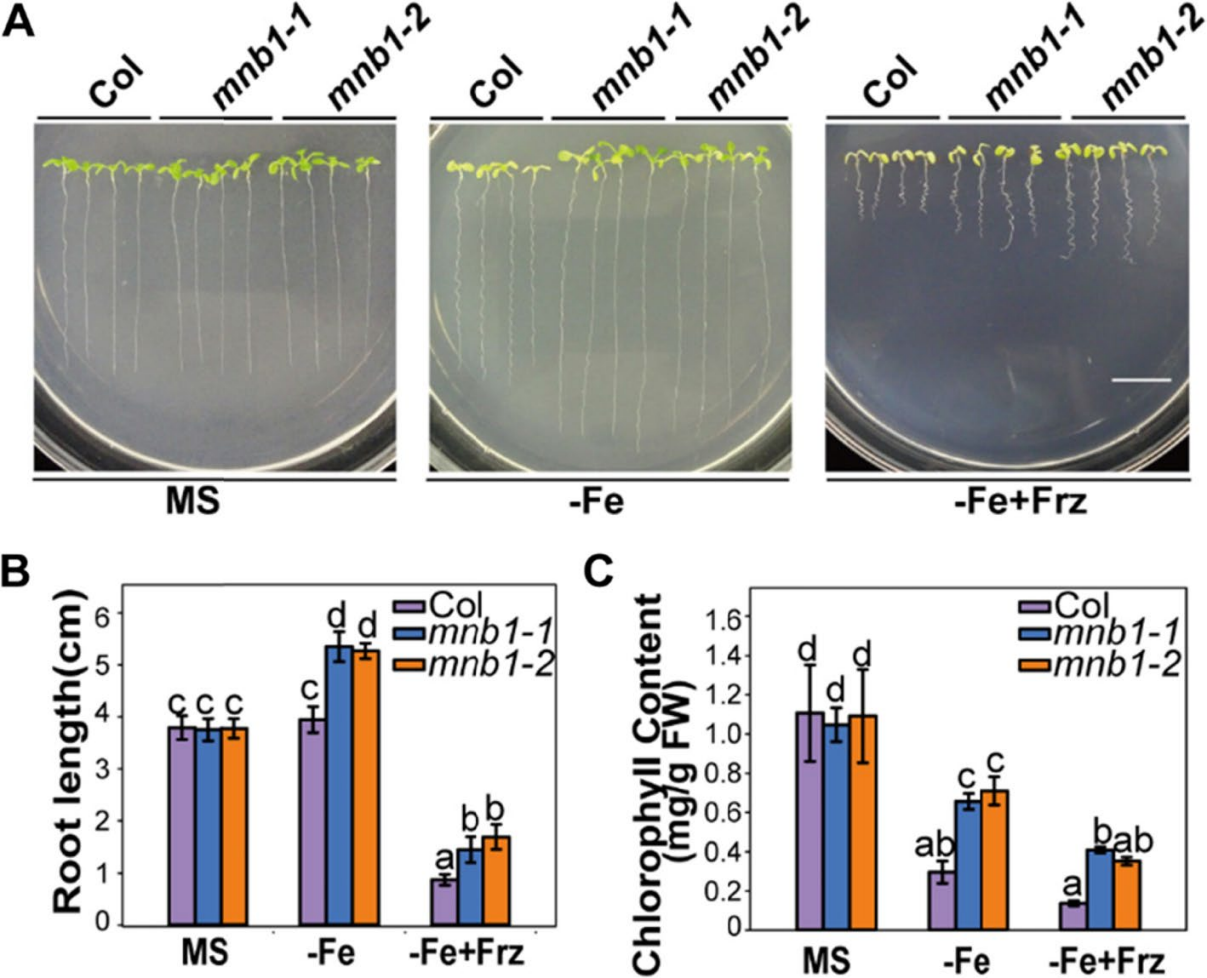
Tolerance of *mnb1* mutant plants to Fe-deficiency stress. **A** Phenotypes of the Col and *mnb1* plants with or without Fe. 3-day old seedlings germinated on MS agar plates were shifted to MS (+Fe, control) or Fe-deficient (−Fe; −Fe + Frz) media for 10 days. Bar = 1 cm. **B, C** Root length (**B**) and total chlorophyll contents (**C**) of Col and *mnb1* mutants under both normal and Fe-deficiency stress conditions were measured. Values are means and SD from three to four independent biological replicates. Statistically significant differences (Tukey’s test, *p* < 0.05) are marked by different lowercase letters

To further determine the role of *MNB1* in the regulation of Fe-deficiency tolerance, we obtained a T-DNA insertion mutant of *MNB1* [28]. qRT-PCR analysis showed that the expression of *MNB1* was not detected in *mnb1* mutant plants during Fe-deficient stress (Additional file 3: Fig.S3A), this result was consistent with our previously study, indicated that the MNB1 function is completely lost due to the T-DNA insertion [28]. After that, we analyzed the response of the loss-of-function *mnb1* mutants to Fe-deficiency stress. We discovered that when the Col and *mnb1* mutant plants were cultivated on MS medium, there was no significant difference between them; whereas *mnb1* mutant plants showed remarkable tolerance when grown in medium without Fe (−Fe), with longer root length and greener leaves than Col (Fig. 1A). The root length and total chlorophyll content of the *mnb1* mutant plants were significantly greater than that of the Col under Fe-limited conditions (Fig. 1B, C). In addition, to further study the role of *MNB1* in the Fe-deficiency stress response, we performed Fe-deficiency tolerance assays in Col, *mnb1-1*, *mnb1-2* plants when cultivated on Fe-deficiency medium (−Fe + Frz, with 50 μM ferrozine to chelate micro metal of Fe from agar) [19], the *mnb1* mutants displayed greater Fe-deficiency tolerance than Col (Fig. 1). Together, these results indicated that the *MNB1* loss-of-function results in increased Fe-deficiency tolerance.

### *MNB1*-overexpressing plants exhibited increased sensitivity to Fe deficiency

**Fig. 2:**
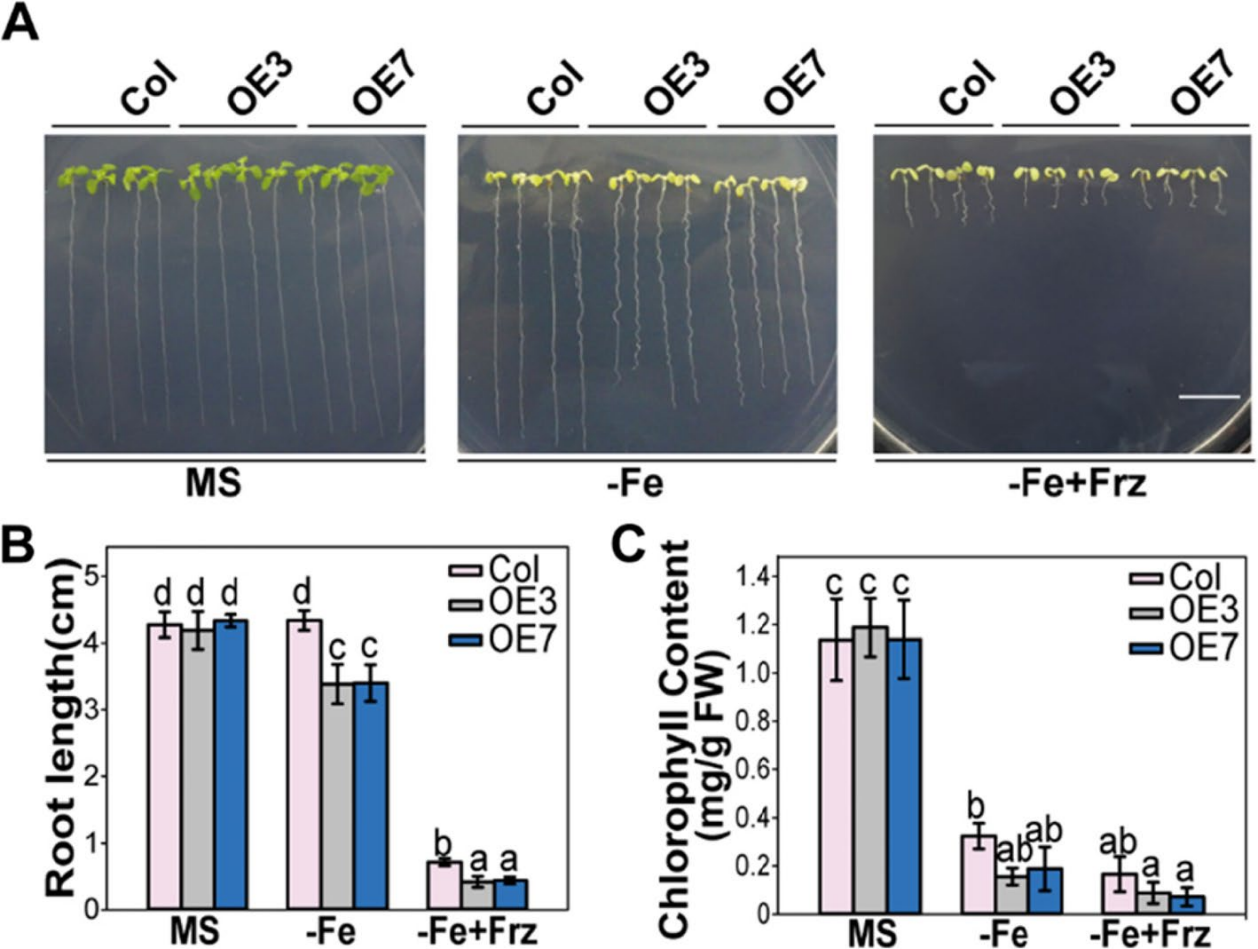
*MNB1*-overexpressing plants exhibit hypersensitivity to Fe-deficient. **A** Phenotypes of the Col, OE3 and OE7 lines with or without Fe. 3-day old seedlings germinated on MS agar plates were shifted to MS (+Fe, control) or Fe-deficient (−Fe; −Fe + Frz) media for 10 days. Bar = 1 cm. **B, C** Root length (**B**) and total chlorophyll contents (**C**) of Col, OE3 and OE7 plants under both normal and Fe-deficiency stress conditions were measured. Values are means and SD from three to four independent biological replicates. Statistically significant differences (Tukey’s test, *p* < 0.05) are marked by different lowercase letters

The role of MNB1 in Fe-deficiency stress response was further confirmed by analyzing the phenotypes of *MNB1*-overexpressing transgenic plants. When the *MNB1*-overexpressing transgenic plants OE3 and OE7 [28] were cultivated on Fe-deficient (−Fe, without Fe) media, they showed significant inhibition of root growth and chlorotic cotyledons compared with that of Col (Fig. 2A). In MS medium, the growth of the Col and OE3 and OE7 lines were not different (Fig. 2A). Under Fe-limited circumstances, the root length and total chlorophyll content of the *MNB1*-overexpressing transgenic plants were lower than that of Col (Fig. 2B, C). Furthermore, we also carried out Fe-deficiency experiments in Col, OE3, OE7 plants when grown on Fe-deficient medium (−Fe + Frz, with 50 μM ferrozine to chelate micro metal of Fe from agar) [19], *MNB1*-overexpressing lines showed hypersensitivity to Fe-deficiency stress (Fig. 2). These results indicated that *MNB1* is important in the regulation of the Fe-deficiency stress response.

### Expression of *MNB1* is repressed by Fe-deficiency stress

**Fig. 3:**
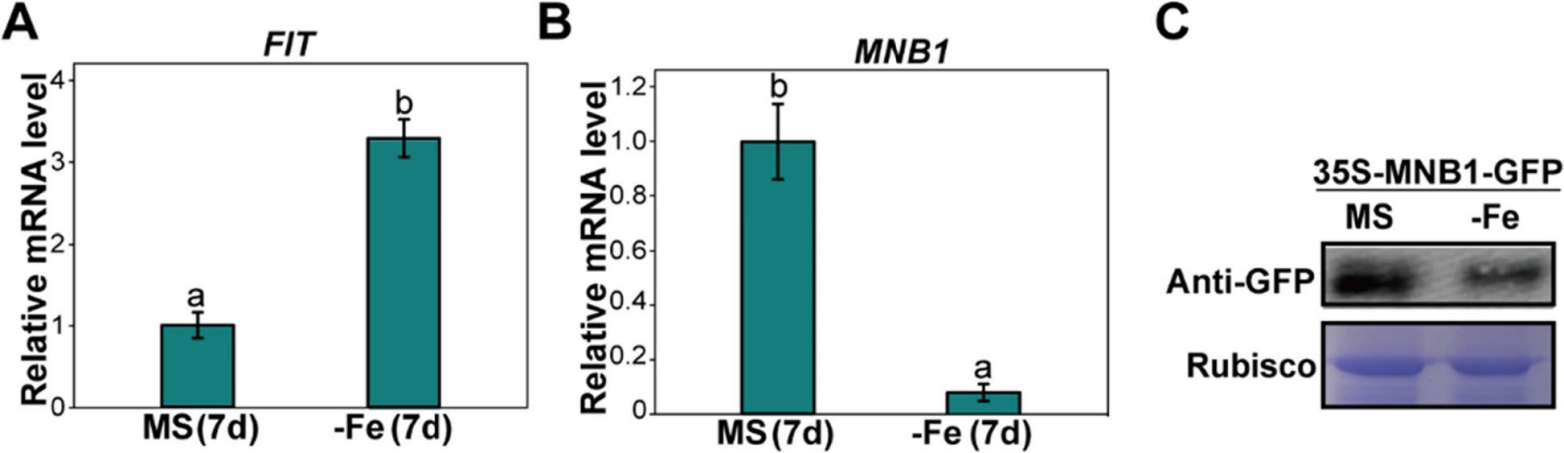
The expression pattern of *MNB1*. **A, B** The expression pattern of *MNB1* was induced under iron-deficient. qRT-PCR analysis of *FIT* and *MNB1* transcript accumulation in the roots of Col. Wild-type plants were germinated on MS agar plate for 10 days and then shifted to MS (+Fe, control) and Fe-deficient (−Fe) for 7 days. *ACTIN8* was used as the internal control. Values are means and SD from three to four independent biological replicates. Statistically significant differences (Tukey’s test, *p* < 0.05) are marked by different lowercase letters. **C** MNB1 protein level under Fe-deficient stress. *35S:MNB1-GFP* transgenic seedlings germinated on MS agar plate for 10 days and then shifted to MS (+Fe, control) and Fe-deficient (−Fe) for 7 days. Protein extracts from treated seedlings and analyzed by 10% SDS-PAGE and Western blot assay. Anti-GFP antibody (upper panel), Rubisco (lower panel) as control. Original images of full-length gels or blots (Additional file 4: Fig. S4)

To evaluate the expression profiles of *MNB1* in Fe-deficiency stress response, the Col seedlings were harvested after treatment for 7 days under conditions of MS and Fe-deficient, and the expression abundance of *FIT* and *MNB1* was analyzed by quantitative real-time PCR. In response to Fe-deficiency stress, the expression of *FIT* was significantly induced (Fig. 3A), whereas the expression of *MNB1* significantly decreased (Fig. 3B). Furthermore, MNB1-GFP transgenic plants were further produced to determine the protein levels of MNB1 in response to Fe-deficiency stress. Western blot results showed that the MNB1 protein accumulation decreased under Fe-deficient conditions (Fig. 3C). These results further confirmed that *MNB1* is involved in the modulation of Fe-deficiency stress response.

### Loss-of-function of *MNB1* reduces ROS level in response to Fe deficiency stress

**Fig. 4:**
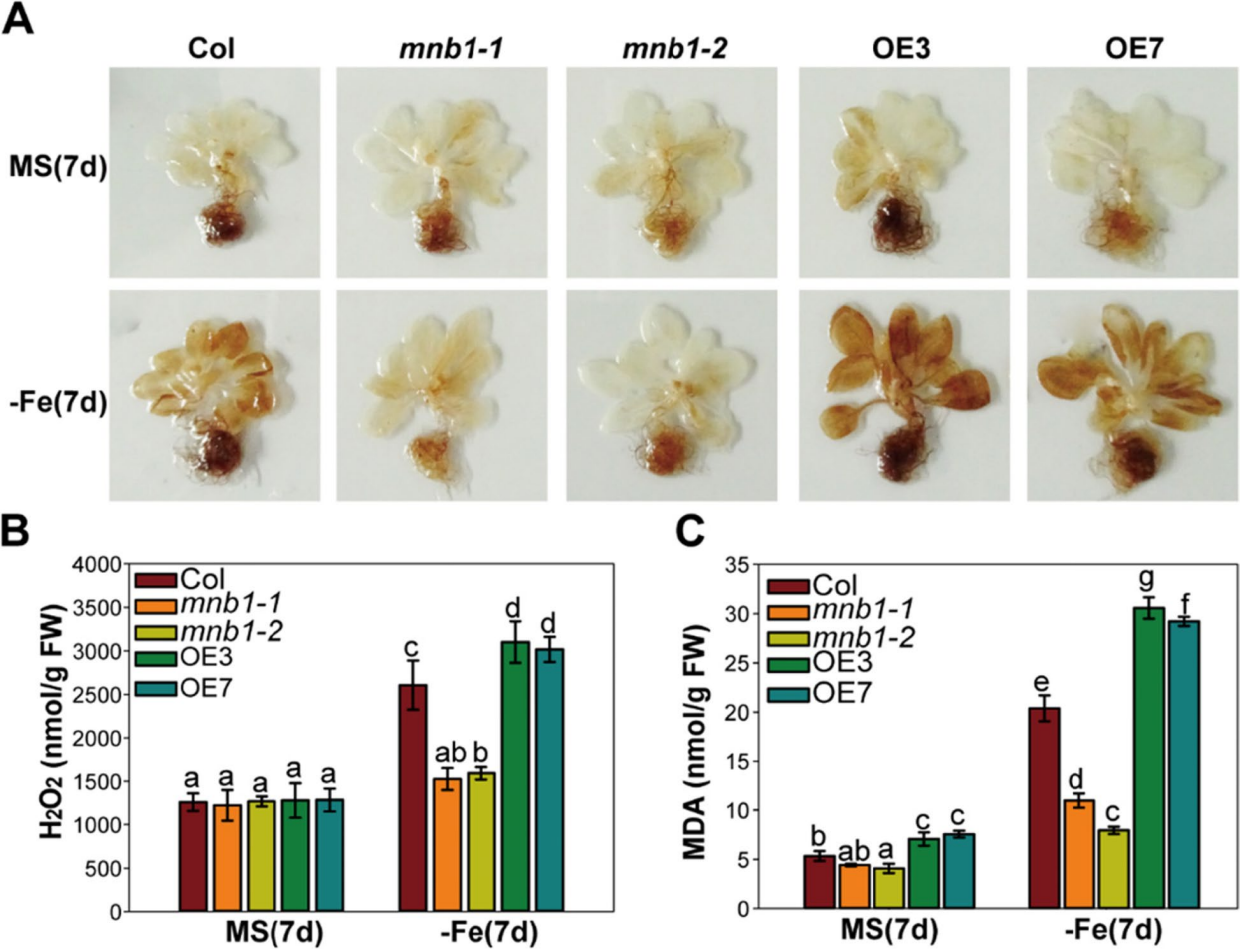
ROS accumulation in the *mnb1* mutants and *MNB1*-overexpressing lines. **A** DAB staining of Col, *mnb1*, OE3, OE7 lines. *Arabidopsis* seedlings germinated on MS agar media for 2 weeks and then shifted to MS (+Fe, control) and Fe-deficient (−Fe) for 7 days. **B** Quantification of H_2_O_2_ from Col, *mnb1-1*, *mnb1-2*, OE3 and OE7 plants. *Arabidopsis* seedlings germinated on MS agar media for 2 weeks and then shifted to MS (+Fe, control) and Fe-deficient (−Fe) for 7 days, and seedling samples were obtained and quantified in H_2_O_2_ concentration. **C** The measurement of MDA in Col, *mnb1-1*, *mnb1-2*, OE3 and OE7 plants. *Arabidopsis* seedlings germinated on MS agar plates for 2 weeks and then shifted to MS (+Fe, control) or Fe-deficient (−Fe) for 7 days, and seedling samples were obtained and quantified in MDA concentration. Values are means and SD from three to four independent biological replicates. Statistically significant differences (Tukey’s test, *p* < 0.05) are marked by different lowercase letters

It was previously indicated that knockout of *CaMBL1* gene resulted in increased disease susceptibility, enhanced bacterial growth, reduced production of ROS in response to an infection with virulent or avirulent Xcv in pepper leaves [24]. Thus, we hypothesized that the loss-of-function of MNB1 decreased the accumulation of ROS under Fe deficiency. To test this hypothesis, we used DAB staining to test changes in ROS level in Col, *mnb1-1*, *mnb1-2*, OE3 and OE7 plants under both normal and Fe-deficiency conditions. Under Fe-deficiency, a less intense staining of DAB was observed in *mnb1* mutant plants as compared with wild-type plants, but a more intense staining of DAB was showed the *MNB1*-overexpressing plants, suggesting lower levels of ROS accumulation in *mnb1* mutant plants, while higher in *MNB1*-overexpressing plants (Fig. 4A). Quantitative measurements of H_2_O_2_ levels under normal conditions showed that, there were no differences in the H_2_O_2_ concentration of the *mnb1* mutants, *MNB1*-overexpressing, and the wild-type plants. However, under Fe-deficiency stress, H_2_O_2_ level was lower in *mnb1* mutants, but higher in *MNB1*-overexpressing plants, when compared to the level in wild-type plants (Fig. 4B).

Due to the fact that ROS leads to cellular oxidative damage in vivo [36], we detected the MDA level in Col, *mnb1-1, mnb1-2*, OE3, OE7 plants under normal and Fe-deficiency conditions. We observed that under Fe-deficiency, the MDA content was lower in the *mnb1* mutant plants and higher in the *MNB1*-overexpressing lines compared with that in Col (Fig. 4C). This indicated a positive correlation between ROS level and MDA content (Fig. 4). The above results indicate that *mnb1* mutant plants encountered reduced oxidative damage under Fe-deficiency stress conditions.

### Knockout of *MNB1* resulted in enhanced expression of genes related to Fe-deficiency under Fe-deficiency stress

**Fig. 5:**
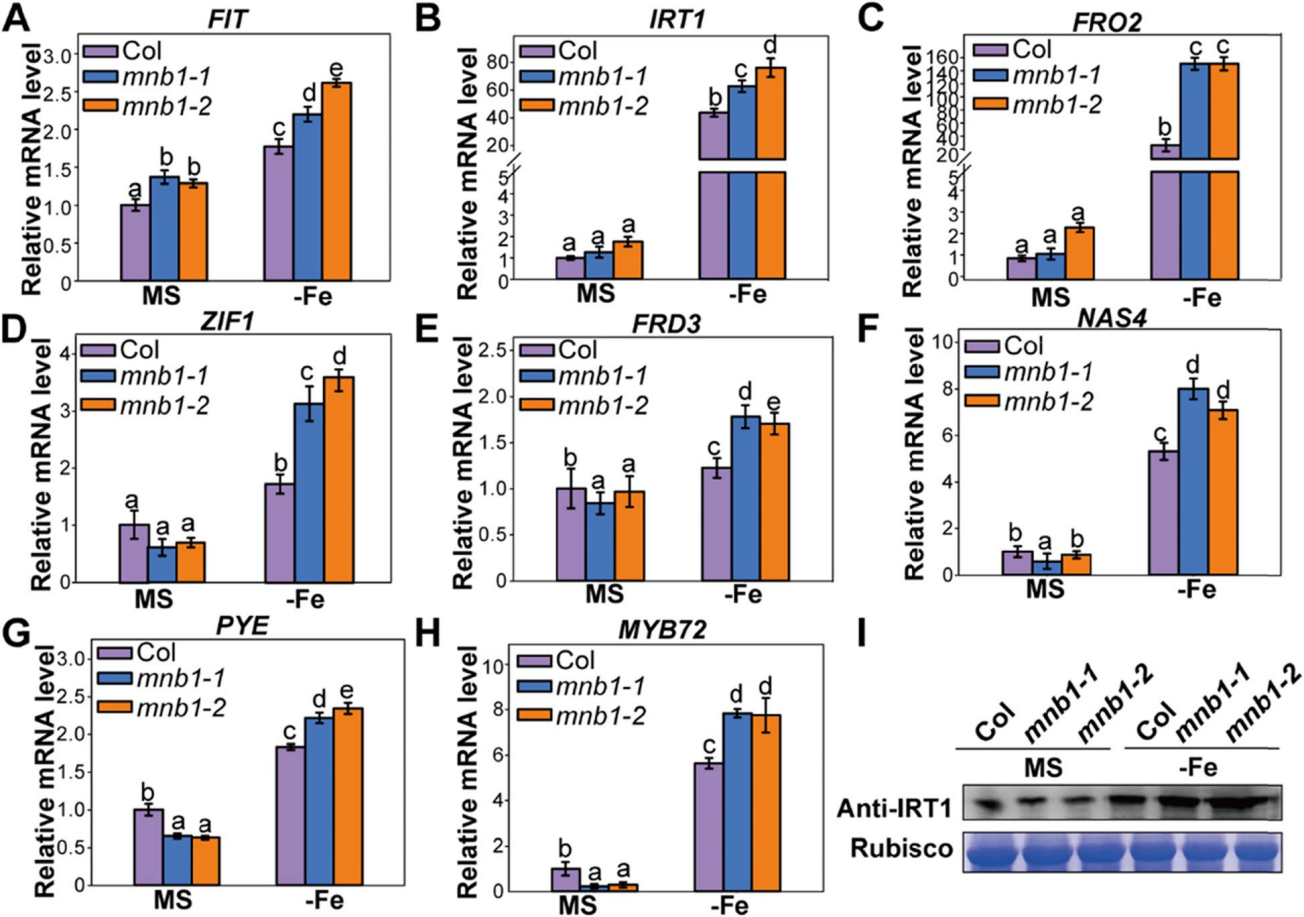
qRT-PCR analysis of the key genes involved in Fe-deficiency stress. **A-H** qRT-PCR analysis of the key genes related to Fe uptake and translocation in the roots of Col and *mnb1* lines. Col and *mnb1* lines were grown vertically on MS agar plate for 10 days and then shifted to MS (+Fe, control) and Fe-deficient (−Fe) for 7 days. *ACTIN8* was used as the internal control. Three independent repeated assays were conducted with similar results, each experiment with three replicates. Values are means and SD from three independent biological replicates. Statistically significant differences (Tukey’s test, *p* < 0.05) are marked by different lowercase letters. **I** IRT1 protein level under Fe-deficient stress. Col and *mnb1* lines grown vertically on MS (+Fe) medium for 10 days and then shifted to MS (+Fe, control) and Fe-deficient (−Fe) for 7 days. Protein extracts from treated seedlings and analyzed by 10% SDS-PAGE and Western blot assay. Anti-IRT1 antibody (upper panel), Rubisco (lower panel) as control. Original images of full-length gels or blots (Additional file 4: Fig. S4)

The above experimental results indicated that the *MNB1* gene effected the ROS levels and may have caused the ROS-mediated signaling, whereas, affecting the expression abundances of Fe-related genes in plants under Fe deficiency. To identify whether *MNB1* had an effect on Fe-related gene expression, we analyzed the relative expression of *Arabidopsis* genes related to Fe uptake and translocation in Col and *mnb1* lines. Under Fe-deficiency stress, transcription levels of several key Fe uptake- and translocation-related genes were examined, including *FIT, IRT1, FRO2, ZIF1, FRD3, NAS4, PYE* and *MYB72* [12–17], and we found the expression of *FIT, IRT1, FRO2, ZIF1, FRD3, NAS4, PYE* and *MYB72* were significantly higher in the *mnb1* mutants in comparison with Col (Fig. 5). Furthermore, the protein level of IRT1 in *mnb1* mutants were further analyzed under Fe deficiency. Western blot results showed that the IRT1 protein accumulation increased under Fe-deficient conditions (Fig. 5I). These findings show that *MNB1*-mediated ROS signaling causes expression of Fe uptake- and translocation-related genes, and thus increased Fe accumulation, which is correlated with enhanced Fe deficiency tolerance.

### Loss-of-function of *MNB1* increases FCR activity

**Fig. 6:**
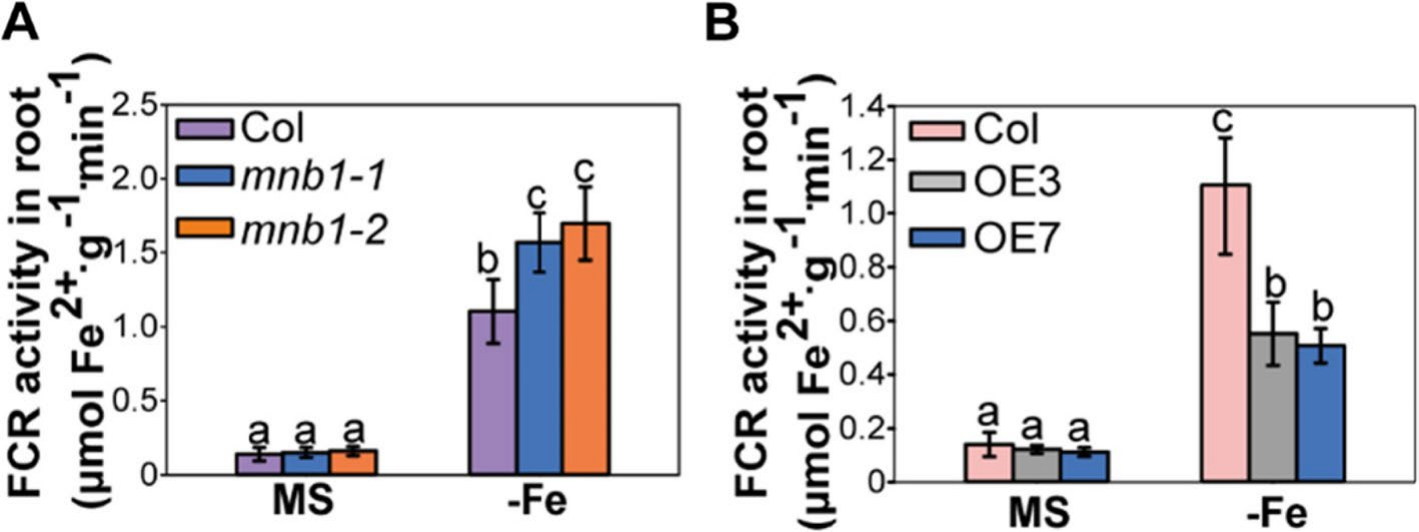
The FCR activity in *mnb1* mutants and *MNB1*-overexpressing plants. **A-B** FCR activity of the Col, *mnb1-1*, *mnb1-2,* OE3 and OE7 plants germinated on MS agar plates for a week and then shifted to MS (+Fe, control) or Fe-deficient (−Fe) media for 3 days. The ferrozine experiment was conducted on 20 pooled plant roots. Values are means and SD from three independent biological replicates. Statistically significant differences (Tukey’s test, *p* < 0.05) are marked by different lowercase letters

Previous studies have shown that FCR activity is also a typical indicator of physiology when Fe is limited, thus we analyzed FCR activity of Col, *mnb1* mutants, and *MNB1*-overexpressing lines using the ferrozine assay under normal and Fe-deficiency stress conditions [37]. We discovered no significant difference in FCR activity between Col, *mnb1* mutants, and *MNB1*-overexpressing plants under normal circumstances. However, under Fe deficiency, the FCR activity of the *mnb1* mutants was significantly higher than that of Col, while the FCR activity of the *MNB1*-overexpressing plants was strikingly lower than that of Col (Fig. 6A, B). Taken together, these results indicate that *MNB1*-mediated Fe-deficiency tolerance is associated with the increased FCR activity.

### Knockout of *MNB1* led to increased Fe concentration level in response to Fe-deficiency stress

**Fig. 7:**
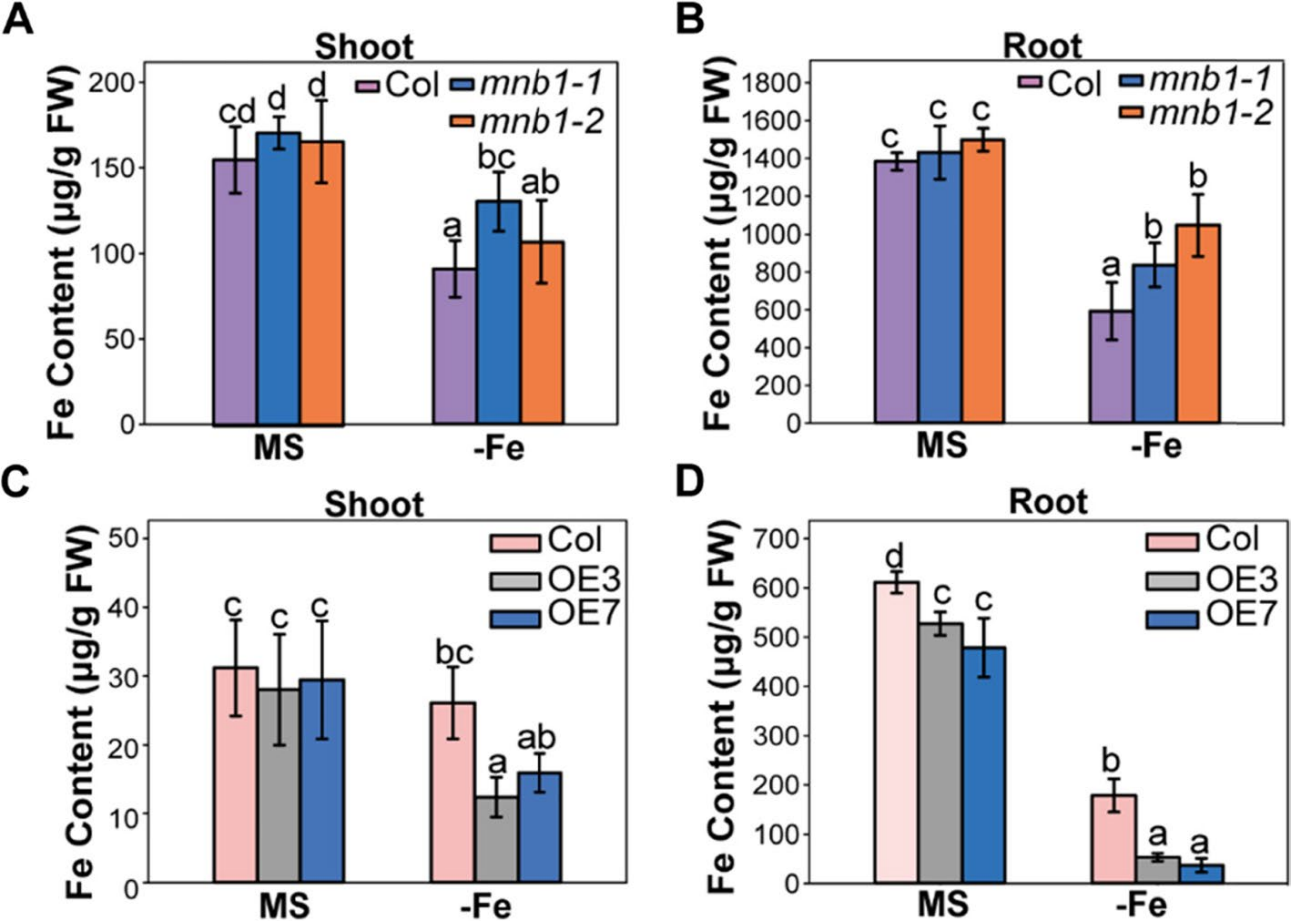
The concentrations of Fe in different plants. **A-D** Fe concentrations in the roots and shoots of Col, *mnb1-1*, *mnb1-2*, OE3 and OE7 plants under Fe-deficient stress. All seedlings were grown vertically on MS (+Fe, control) and Fe-deficient (−Fe) for 10 days. Values are means and SD from three independent biological replicates. Statistically significant differences (Tukey’s test, *p* < 0.05) are marked by different lowercase letters

*FIT*, *IRT1* and *FRO2* genes play a vital role in iron acquisition in plants [38, 39]. The above results proved that the expression levels of Fe uptake- and translocation-related genes increased in the *mnb1* mutants than Col under Fe deficiency. Therefore, we measured the Fe concentration of Col, *mnb1* mutants, and *MNB1*-overexpressing plants under both normal and Fe-deficiency conditions. Under Fe-deficiency stress conditions, we found that the Fe content was strikingly higher in the leaves and roots of the *mnb1* mutants compared with that Col (Fig. 7A, B), while the Fe concentration in the leaves and roots of the OE3 and OE7 lines were strikingly lower than that Col (Fig. 7C, D). In addition, we also measured the Fe concentration of Col, *mnb1* mutants, and *MNB1*-overexpressing seeds under both normal and Fe-deficiency conditions. we found that the Fe concentration was higher in seeds of the *mnb1* mutants compared with that in the Col, while the Fe content in seeds of the *MNB1*-overexpressing were lower than that in the Col (Additional file 3: Fig.S3B). These results further support that *MNB1*-mediated Fe accumulation and tolerance were consistent with increased expression of Fe-uptake genes (Fig. 5).

## Discussion

Fe is an indispensable mineral element for normal growth of plants. Fe shortage leads to delayed growth and decreased photosynthesis, resulting in lower crop production. To cope with the threat of a Fe-limited environment, plants may sense external Fe status and utilize complicated mechanisms to modulate the expression of Fe uptake-related genes, thereby facilitating Fe influx from soils in order to satisfy the plant’s requirements for Fe. On the other hand, Fe-overload is harmful to plants, owing to the generation of hydroxyl radicals (OH˙) via Fenton reaction, which is the most active ROS [33, 40]. Thus, in plants, it is really key to maintain the homeostasis of Fe. Plants have developed complicated regulatory networks to modulate Fe homeostasis and deficiency responses. However, the molecular regulatory mechanisms for Fe-deficiency stress response are still not well understood in plants, and only a number of Fe deficiency-responsive genes have been identified and characterized [12–14, 21]. In previous research, mannose-binding lectins play important roles in many biological processes of plants, including defense signaling response during pathogen attack, and plant hormone responses by specifically binding to carbohydrates [24, 41, 42]. It has been reported that the association of pepper mannose-binding lectin CaMBL1 with mannose may play a crucial role in modulating cell death and defense responses against microbial pathogens attack [24]. To our knowledge, so far, whether plant mannose-binding lectins modulates iron-deficient responses or not, and how it occurs, remain uncertain. In the present research, we found that the Arabidopsis mannose-binding lectin *MNB1* negatively modulates the Fe accumulation and tolerance under Fe-deficient conditions. According to the evidence presented here, *MNB1* transcription was severely repressed, and MNB1 protein accumulation decreased in response to Fe shortage stress (Fig. 3B, C). Additionally, knockout of *MNB1* displayed enhanced tolerance to Fe-deficient, including increased the plant root length, leaf chlorophyll content, and FCR activity (Figs. 1 and 6), whereas overexpression of *MNB1* plants showed sensitivity phenotype to Fe-deficient, including decreased plant root length, FCR activity, and chlorotic leaves (Figs. 2 and 6).

ROS homeostasis is widely recognized to promote cellular development and proliferation as a result of positive ROS signaling; nevertheless, excessive ROS production causes oxidative stress, which can lead to cellular damage or even death [43]. Some early studies have indicated that higher amounts of ROS are generated in defective and permeable cuticles under pathogen attack, which play a role in resistance against viral attack [44, 45]. A recent study showed that reduced antioxidant activity, higher ROS accumulation and advanced cell death in the *dps1* mutant contribute to panicle apical degeneration and fertility reduction [30]. In addition, higher accumulation of ROS can also lead to male sterility phenotypes such as defective anther development, aborted pollen grains and failure of fertilization [30, 32]. Several studies have reported the ROS-mediated increased cell death in different vegetative and reproductive tissues [45, 46]. Increasing evidence indicates that ROS is an important signaling molecule and regulates the expression of various genes [47]. The study by Sun et al. [48] indicated that ROS mediates between positive and negative modulation of plant responses to Fe-deficiency stress and ROS participates in Fe distribution in roots. Under Fe shortage stress, we found that the total Fe concentration of roots and leaves in both *mnb1* mutants and *MNB1-OE* lines was significantly different from Col (Fig. 7). *MNB1* or ROS may be participated in Fe distribution between roots and shoots, based on this finding. Furthermore, previous research shows that the silencing of pepper mannose-binding lectin led to increased disease susceptibility, enhanced bacterial growth, reduced production of ROS in response to an infection with virulent or avirulent Xcv in pepper leaves [24]. As a result, we hypothesized that in *Arabidopsis*, the analogous mannose-binding protein *MNB1* would affect plant responses to Fe shortage stress via ROS-mediated signaling. MDA levels in Col, *mnb1-1*, *mnb1-2* and *MNB1-OE* lines under treatments with or without Fe confirmed this notion concerning *MNB1* participation in our investigation (Fig. 4C). Surprisingly, we found that ROS level in *mnb1* mutants was lower than Col in Fe-deficiency treatments, whereas ROS level in *MNB1*-overexpressing plants was higher than Col in Fe-deficiency treatments (Fig. 4A, B). These findings suggest that *MNB1* may regulate the Fe-deficiency stress response by affecting the endogenous ROS level. ROS plays a critical part in the complex communication networks that activate defensive systems when a plant is exposed to biotic and abiotic stresses [49]. It was reported that proteins embedded in plasma membranes a have been discovered to be part of a monitoring system that is needed for the recognition and transduction of defense-related signals in plant [50]. Recently, a study reported that *MNB1* is a membrane-associated protein [28]. Therefore, we speculated that *MNB1* would exert an important role in signal transduction. However, the biochemical function of *MNB1* needs further study in future research. Stress caused by a lack of Fe impacted *MNB1* transcription level and thereby altered the quantity of ROS and ROS-mediated signaling, as a result, effecting the expression of Fe-related genes in the nucleus. In response to Fe shortage stress, plants have developed a number of adaptive molecular mechanisms, the most notable of which are the Fe absorption and translocation routes [38, 39]. According to earlier studies, ROS-mediated signaling is transduced to the nucleus, which changes the expression patterns of the nuclear genes correlated to Fe-deficient stress [51], such as *FIT*, *IRT1,* and *FRO2* to regulate Fe-deficiency tolerance. As a result, we checked the transcription level of the genes related to Fe-deficient-stress. The silencing of *MNB1* increased the expression abundances of *FIT, IRT1, FRO2, ZIF1, FRD3, NAS4, PYE and MYB72* during Fe-deficient stress (Fig. 5). Our results revealed that the *MNB1* gene regulated Fe-deficient stress by effecting expression abundances of Fe uptake- and translocation-related genes.

In the present work, we also investigated whether *MNB1* is participated in modulating other abiotic stress responses, such as MnSO_4_, High Fe, and H_2_O_2_, and discovered that the growth of *mnb1-1* and *mnb1-2* mutants were strikingly different from that of Col under MnSO_4_, high Fe and H_2_O_2_ stresses, indicating that *MNB1* may also be participated in the modulation of MnSO_4_, high Fe and H_2_O_2_ stresses responses. Consequently, investigating the molecular mechanisms of MNB1-mediated MnSO_4_, High Fe, and H_2_O_2_ stresses may be interesting (Additional file 1: Fig. S1). Furthermore, owing to MNB1 proteins have been reported to be able to bind to d-Mannose, we studied whether the mannose is required for MNB1-mediated Fe deficiency tolerance [28]. To illustrate this hypothesis, we used exogenous mannose to deal with Col, *mnb1-1*, *mnb1-2*, OE3 and OE7 plants, and found that no notable differences were observed to exogenous mannose was added with Fe-limited media (Additional file 2: Fig.S2). This may suggest that the mannose is not required for MNB1-mediated Fe deficiency tolerance.

**Fig. 8:**
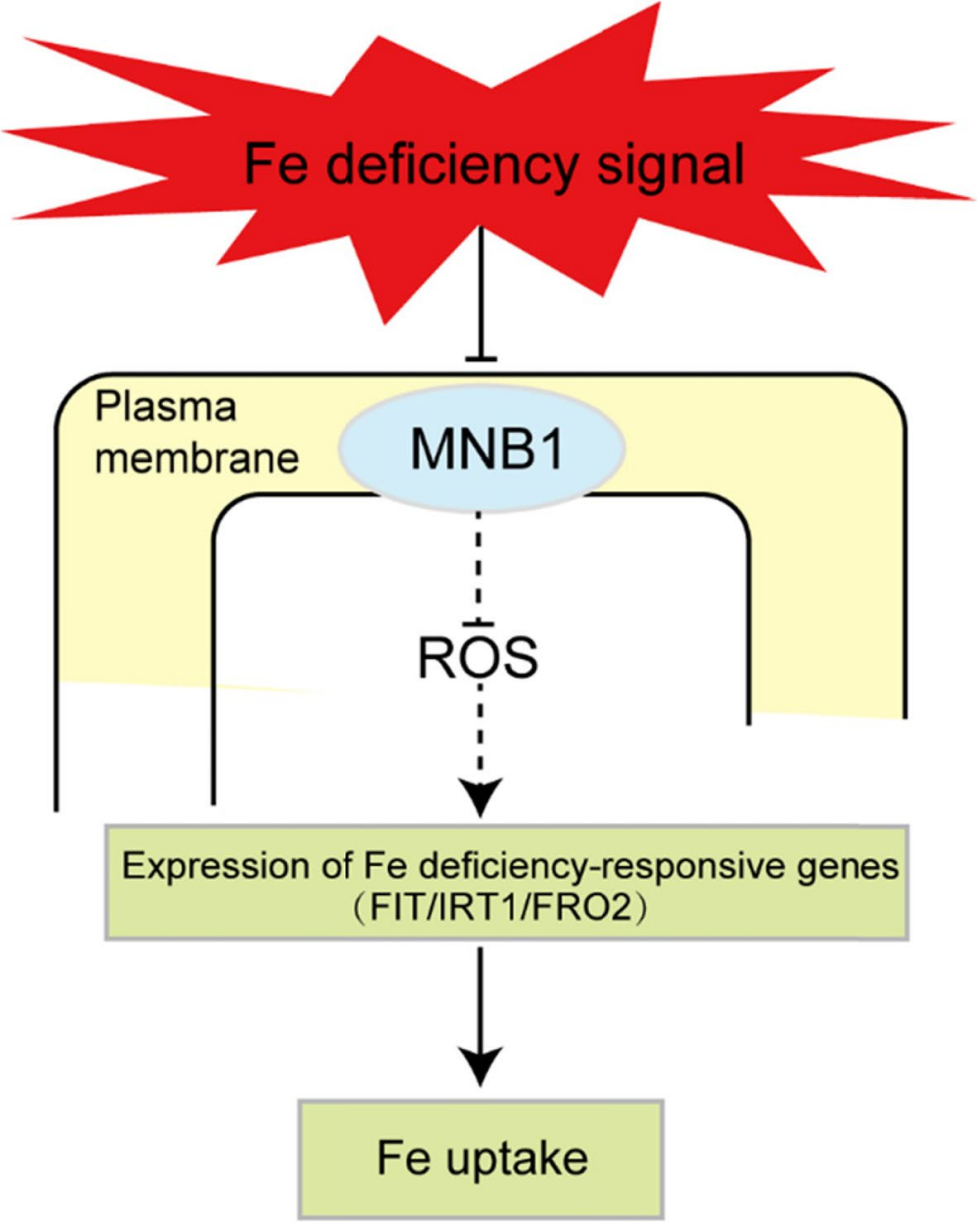
A model for the role of *MNB1* gene in modulating Fe-deficient tolerance in *Arabidopsis*. Fe-deficient stress inhibited the expression of *MNB1* gene, which leads to an increase the expression of Fe uptake and translocation gene through affecting ROS level and signaling, and thus enhanced the accumulation of Fe content in *Arabidopsis*

In conclusion, our findings shed light on the biological functions of mannose-binding lectins in plants. Plants’ iron-deficiency stress tolerance is regulated by *MNB1*. Fe-deficiency stress inhibits the expression pattern of *MNB1*, thereby decreasing ROS levels and changing ROS-modulated signaling. This resulted in increased expression abundances of Fe uptake- and translocation-related genes (*FIT, IRT1, FRO2, ZIF1, FRD3, NAS4, PYE and MYB72*), increased Fe accumulation, and enhanced Fe-deficiency tolerance (Fig. 8).

## Methods

### Plant materials, growth conditions, and treatments

In this work, the *A. thaliana* wild-type (Col), *mnb1-1* (SALK-038821C)*, mnb1-2* (SALK-121641) and the transgenic lines overexpressing *MNB1* have been described previously [28]. *Arabidopsis* mutants were obtained from the Arabidopsis Biological Resource Centre (ABRC) at Ohio State University, USA. *Arabidopsis* seeds were surface sterilized in 0.1 M HgCl_2_, and washed three times with sterile distilled water (ddH_2_O) to remove 0.1 M HgCl_2_ solution, and then cultured on Murashige and Skoog (MS media, Caisson, USA) nutrient medium, supplemented with 1% sucrose (w/v) and 1.2% (w/v) agar, adjusted to pH 5.8. Culture plates were vernalized for 3 days in a refrigerator in darkness at a temperature of 4 °C and then placed into a culture room at 22 °C, under a light intensity of 100 μmol m^− 2^ s^− 1^ and a long day (16 h of light/8 h of dark cycles).

For phenotypic analyses of the Fe-deficient-stress response, seeds of Col, *mnb1-1*, *mnb1-2*, OE3, and OE7 [28] plants were germinated on MS media for 3 days, and then transferred to +Fe [MS media as a control] or Fe-deficiency medium [−Fe, without Fe(II)-EDTA; −Fe + Frz, with 50 μM ferrous chelate ferrozine (Sangon Biotech, China)]. After 10 days of growth, the plants were photographed and then subjected to growth experiments, and their root length and total chlorophyll content were measured. All experiments were carried out three times independently, and more than 30 *Arabidopsis* seedlings used in each measurement. For Fe-deficient inducible gene expression analysis, the *Arabidopsis* seedlings were cultured for 10 days on MS medium and then shifted to MS(+Fe) or –Fe medium for 7 days, finally, these plant materials were used for further analysis. For seeds Fe content measurement, all plants were grown in the float to uptake the +Fe [MS solution as a control] or Fe-deficiency solution [−Fe, without Fe(II)-EDTA], and harvested dry seeds after 8 weeks. Finally, these seed materials were used for further analysis.

### Generation of 35S:MNB1-GFP transgenic plants

To generate 35S:MNB1- green fluorescent protein (GFP) transgenic plants, the protein coding region of *MNB1*(AT1G78830) was amplified from *Arabidopsis* with specific primers by PCR (Additional file 5: Table S1), digested with Kpn1 and Xho1 restriction enzymes (called *35S:MNB1*-GFP), and then cloned into the *pART27* vector containing Cauliflower mosaic virus 35S promoter and GFP reporter. The *35S:MNB1-GFP* recombinant vectors were introduced into the *Agrobacterium tumefaciens* GV3101 strain and then transformed into *Arabidopsis* wild-type lines by using the floral dip method [52]. The *35S:MNB1-GFP* lines were T3 homozygous plants used in this work. All the obtained transgenic lines were chosen for further experiments.

### RNA extraction and real-time quantitative RT-PCR (qRT-PCR) analysis

Total RNA of whole seedlings was extracted using Trizol Reagent (Invitrogen, Life Technologies, USA) following the manufacturer’s protocols and then used to synthesize cDNA. Reverse transcription reactions were performed as described previously [53]. qRT-PCR was carried out in the Bio-Rad iCycler iQ system (Bio-Rad Laboratories, USA) applying a TransStart Tip Green qPCR SuperMix (Transgen, Beijing, China) following the manufacturer’s method. *ACTIN8*(AT1G49240) was used as the internal control. All experiments were carried out at least in triplicate. The specific qRT-PCR primers used are listed in Additional file 5: Table S1.

### 3,3′-Diaminobenzidine (DAB) staining

DAB staining was performed according to an adaptation of a previously reported protocol [54]. Specifically, *Arabidopsis* seedling samples were obtained and vacuum-immersed in 3,3′-diaminobenzidine solution (DAB, 1 mg/mL, pH 3.8, Sigma-Aldrich) for 15 min and before being incubated in a gyratory shaker at 25 °C for 3-4 h. Following the termination of the DAB staining reaction, these samples were fixed in a bleaching buffer solution (ethanol/ glycerol / acetic acid = 3:1:1). After samples were photographed using camera [55].

### Protein extraction and western blotting assay

Total protein of *Arabidopsis* seedlings was extracted and boiled as previously described [56]. Briefly, proteins were separated by SDS-polyacrylamide gel electrophoresis (PAGE). After transferring to poly (vinylidene fluoride) (PVDF) membranes and blocking with 4% nonfat milk, immunoblot was probed using specific antibodies. The plant antibodies were purchased from PhytoAB company (California, USA). Western Blotting Detection System was conducted using an EasySee Western Blot Kit (Sangon Biotech, China).

### Malondialdehyde (MDA) measurement

Determination of malondialdehyde (MDA) content in *Arabidopsis* seedlings through the thiobarbituric acid reaction as used the protocol described by Hodges et al., (1999) [57]. wild-type, *mnb1-1*, *mnb1-2*, OE3, and OE7 plants were obtained and homogenized in 80% (v/v) ethanol. The homogenate solution was centrifuged at 11,500×g for 10 min, and the supernatant was remained and added with 1.5 mL 5 % trichloroacetic acid (TCA) containing different concentrations of thiobarbituric acid. The reaction mixture was then heated at a temperature of 95 °C with a water bath for about 30 min and then rapidly cooled in an ice-water bath. The spectrophotometric absorbance was monitored at 450, 532, and 600 nm. After that, the concentration of MDA was calculated according to the parameters of various dilutions of reference solutions.

### Hydrogen peroxide (H_2_O_2_) measurement

ROS was measured in terms of H_2_O_2_ following the instructions provided by hydrogen peroxide assay kit (Sangon Biotech, China). Briefly, 0.1 g *Arabidopsis* seedlings were extracted with 1 ml acetone and centrifuged for 10 min at 8000 g at 4 °C. The supernatant was used to measure OD at 415 nm. H_2_O_2_ was then estimated from standard curve.

### Ferric Chelate Reductase (FCR) assays

The measurement of FCR activity was carried out according to a previous study [37]. Briefly, fresh 20 whole seedlings of each sample pretreated for about 30 min in glass plates with 4 mL of MS buffer solution absence micro-nutrients at pH 5.5 (pH adjusted by addition of HCl) and then immersed with 5 mL of Fe (III) reduction assay buffer solution [MS buffer solution absence micro-nutrients, 0.3 mM ferrozine, and 0.1 mM Fe (III)-EDTA, (at pH 5.0)] for about 40 min in the darkness. An identical experiment buffer-solution without samples was used as a blank. The absorbance of the Fe(II)-ferrozine complex was measured at 562 nm.

### Determination of total chlorophyll content

Total chlorophyll was extracted from two-week-old seedlings in darkness at a room temperature using 80% acetone. At 645 nm and 663 nm, the supernatant was spectrophotometrically analyzed. The total chlorophyll content was determined modifying the protocol of Aono et al., (1993) [58].

### Fe concentration measurement

For Fe concentration measurement, *Arabidopsis* seeds were grown on the MS or Fe-deficient media for 10 days. The sample of root and shoot tissues were collected separately and used for the analysis of Fe content measurement. For seeds Fe content measurement, all plants were grown in the float to uptake the +Fe [MS solution as a control] or Fe-deficiency solution [−Fe, without Fe(II)-EDTA], and harvested dry seeds after 8 weeks. These seeds were used for the analysis of Fe content measurement. After that, all samples were dried at 100 °C for 40 min and 80 °C for 2 days, weighed, and digested in a mixture of concentrated 10% perchloric acid and 30% nitric acid in a microwave digestion system ETHOS1 (Milestone). Three samples were used for Fe content measurement in each independent experiment, and Fe concentrations were measured by ICPOES (model 5300DV; PerkinElmer, USA), as described previously [59].

## Accession numbers

Sequence data from this article can be found in the *Arabidopsis* Genome Initiative or GeneBank / EMBL database under the following accession numbers: *MNB1* (AT1G78830)*, FIT* (AT2G28160)*, FRO2 (AT1G01580*)*, IRT1* (AT4G19690)*, ZIF1* (At5G13740)*, FRD3* (At3G08040)*, NAS4* (AT1G5643*0), PYE* (At3G47640)*, MYB72* (At1G56160) and *ACTIN8* (AT1G49240).

## Supplementary Information

**Additional file 1: Figure S1.** Phenotype of *mnb1* mutants under many other abiotic stresses.

**Additional file 2: Figure S2.** Tolerance of Col, *mnb1* mutants and *MNB1*-overexpressing lines to mannose or Fe-deficient stress.

**Additional file 3: Figure S3.** Identification of mnb1 mutant materials and determination of the Fe concentration of Col, *mnb1* mutants, and *MNB1*-overexpressing seeds.

**Additional file 4: Figure S4.** Original images of full-length gels or blots.

**Additional file 5: Table S1.** Primers used for cloning and qRT-PCR assay.

## Data Availability

The datasets used and/or analyzed during the current study are available from the corresponding author on reasonable request.
