# Supplementary material for: MNB1 gene is involved in regulating the iron-deficiency stress response in Arabidopsis thaliana
Source: BMC Plant Biol. 2022 Mar 28;22:151. doi: 10.1186/s12870-022-03553-5 (PMC8961904; doi:10.1186/s12870-022-03553-5)
Supplement: Supplementary file 1 — Additional file 1: Figure S1. Phenotype of mnb1 mutants under many other abiotic stresses. [file 12870_2022_3553_MOESM1_ESM.docx]

***MNB1* gene is involved in regulating the iron-deficiency stress response in *Arabidopsis thaliana***

Hui Song^1,2*^, Feng Chen^1,2*^, Xi Wu^1,2*^, Min Hu^1,2^, Qingliu Geng^1,2^, Min Ye^1,2^, Cheng Zhang^1,2^, Li Jiang^1,2§^ and Shuqing Cao^1,2§^

Engineering Research Center of Bio-process, Ministry of Education, ^2^School of Food and Biological Engineering, Hefei University of Technology，Hefei 230009, China.

*These authors contributed equally to this work.

^§^Corresponding author: shuqingcao@hfut.edu.cn or jiangli@ustc.edu.cn.


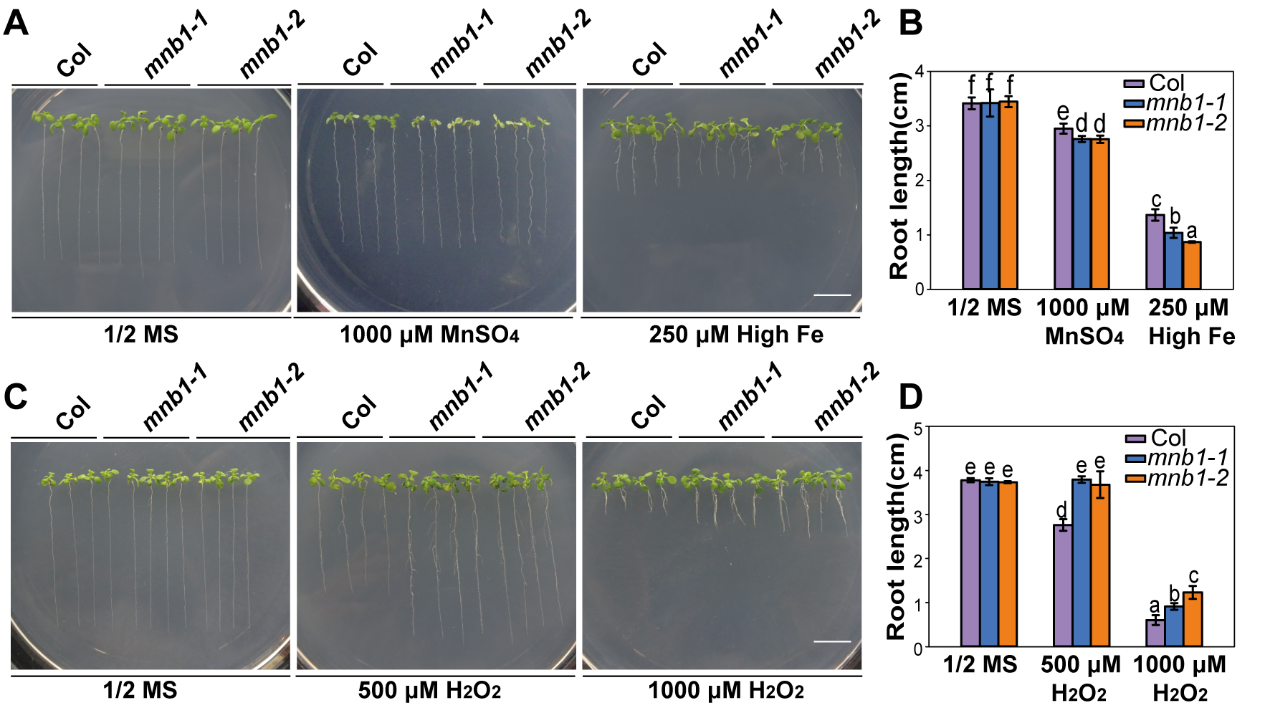


**Fig. S1.** Phenotype of *mnb1* mutants under many other abiotic stresses. (A, C) Tolerance of Col, *mnb1-1* and *mnb1-2* plants to 1000 μM MnSO_4_, 250 μM High Fe, 500 μM H_2_O_2_ or 1000 μM H_2_O_2_ stresses. 3-day old seedlings germinated on 1/2 MS agar plates were shifted to 1/2 MS media with or without 1000 μM MnSO_4_, 250 μM High Fe, 500 μM H_2_O_2_ or 1000 μM H_2_O_2_ for about 2 weeks. Bar = 1 cm. (B, D) Root length (B, D) of Col, *mnb1-1* and *mnb1-2* plants under normal and1000 μM MnSO_4_, 250 μM High Fe, 500 μM H_2_O_2_ or 1000 μM H_2_O_2_ stresses were measured. Values are means and SD from three independent biological replicates. Statistically significant differences (Tukey’s test, *p* < 0.05) are marked by different lowercase letters.


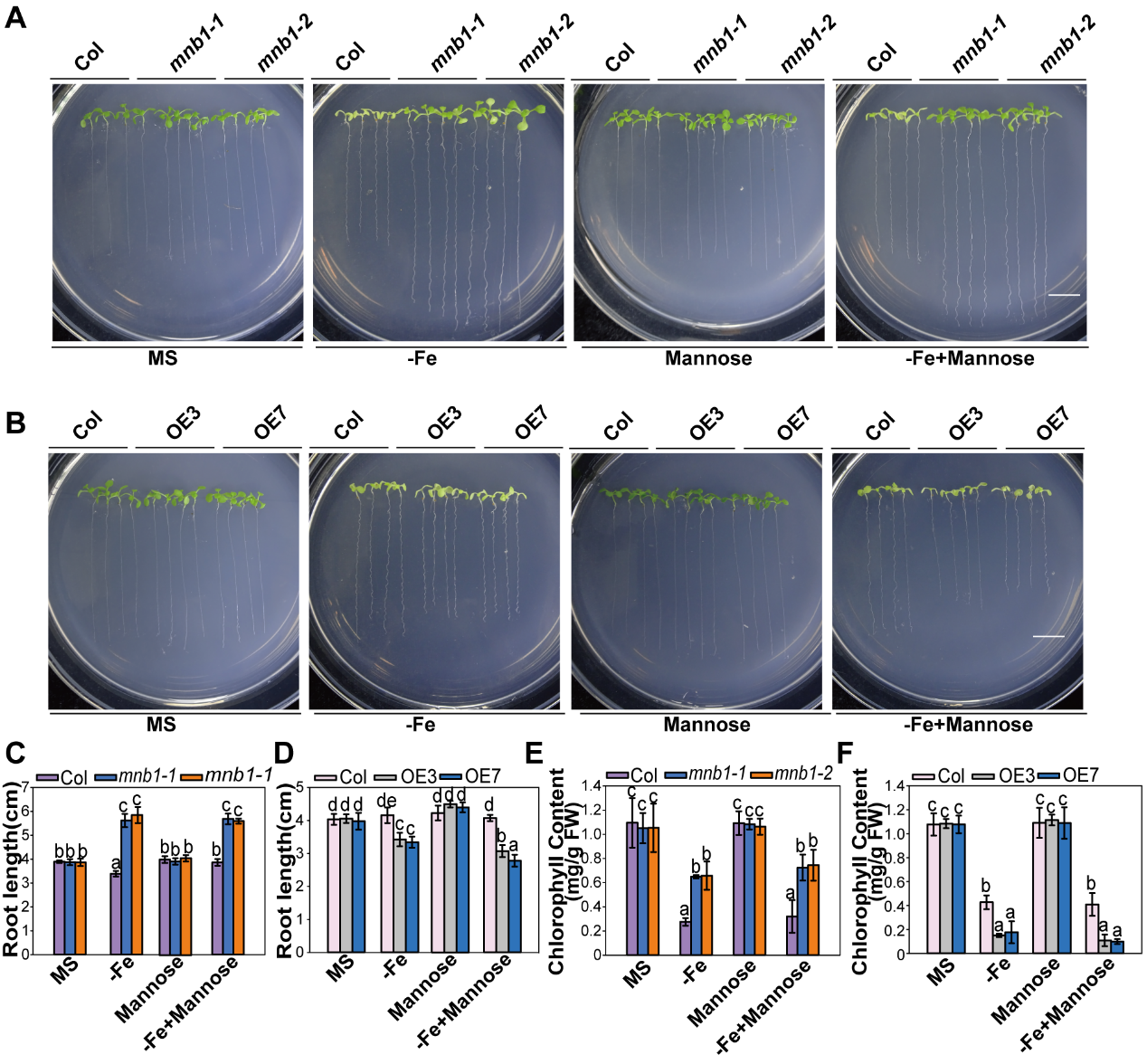


**Fig. S2.** Tolerance of Col, *mnb1* mutants and *MNB1*-overexpressing lines to mannose or Fe-deficient stress (A-B) Effect of 1.5 mM mannose treatment on Fe-deficient tolerance of the Col, *mnb1-1*, *mnb1-2,* OE3 and OE7 plants with or without Fe. 3-day old seedlings germinated on MS (+Fe) agar plates were shifted to MS media with or without Fe or 1.5 mM mannose for about 10 days. Bar = 1 cm. (C-F) Root length (C, D) and total chlorophyll content (E, F) of Col, *mnb1-1*, *mnb1-2,* OE3 and OE7 plants under both normal and Fe-deficiency stress conditions were measured. Values are means and SD from three independent biological replicates. Statistically significant differences (Tukey’s test, *p* < 0.05) are marked by different lowercase letters.


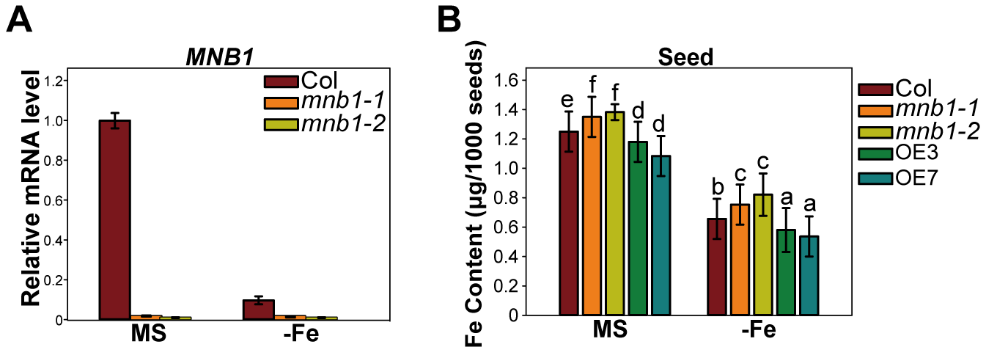


**Fig. S3.** Identification of *mnb1* mutant materials and determination of the Fe concentration of Col, *mnb1* mutants, and *MNB1*-overexpressing seeds. (A) qRT-PCR analysis of *MNB1* transcript level in Col and *mnb1* mutants under normal and Fe-deficiency conditions. *ACTIN8* was used as an internal control. Data are presented as means ±SE of three biological replicates. All seedlings were grown vertically on MS agar plate for 10 days and then shifted to MS (+Fe, control) and Fe-deficient (-Fe) for 7 days. Values are means and SD from three independent biological replicates. Statistically significant differences (Tukey’s test, *p* < 0.05) are marked by different lowercase letters. (B) Fe concentrations in the seeds of Col, *mnb1-1*, *mnb1-2*, OE3 and OE7 plants under normal and Fe-deficiency stress. Values are means and SD from three biological replicates of 1000 seeds. Statistically significant differences (Tukey’s test, *p* < 0.05) are marked by different lowercase letters.


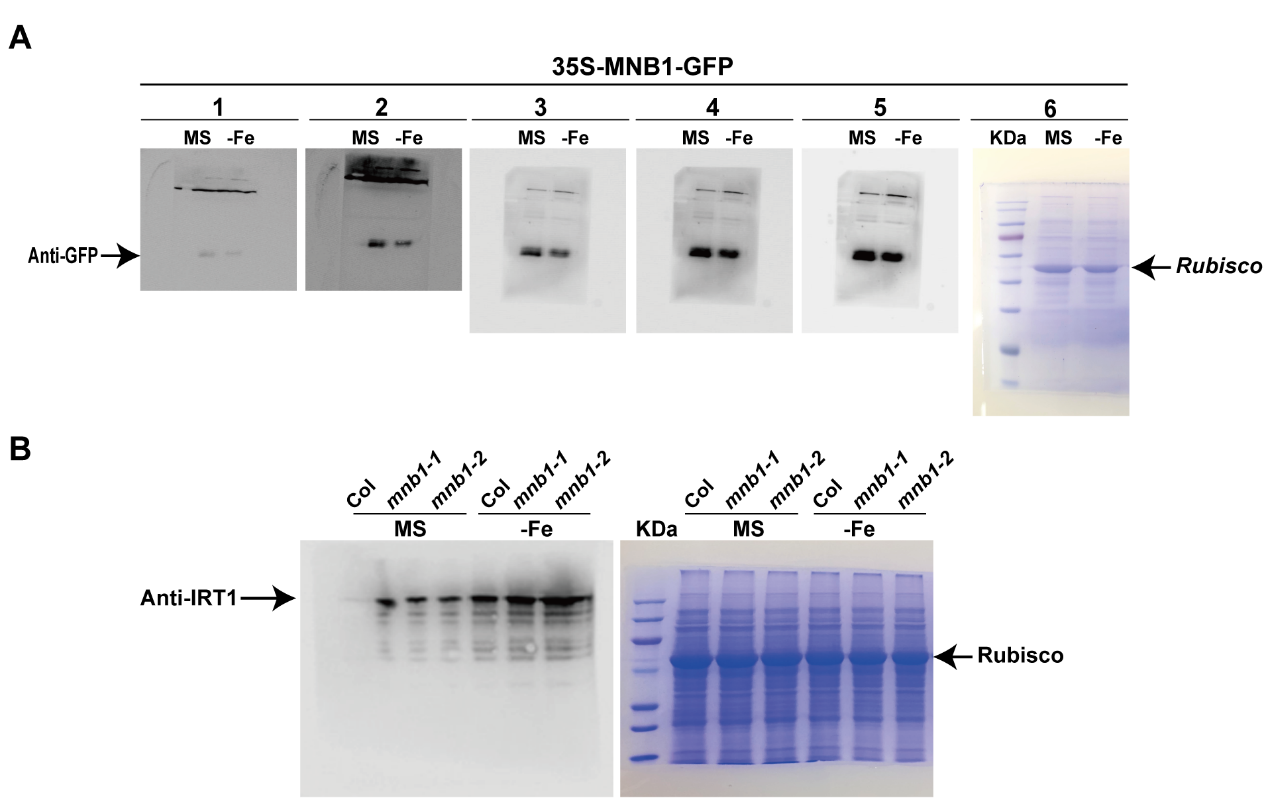


**Fig. S4.** Original images of full-length gels or blots. (A) corresponds to Fig 3C. MNB1 protein level under Fe-deficient stress. *35S:MNB1-GFP* transgenic seedlings germinated on MS agar plate for 10 days and then shifted to MS (+Fe, control) and Fe-deficient (-Fe) for 7 days. Protein extracts from treated seedlings and analyzed by 10% SDS-PAGE and Western blot assay. Anti-GFP antibody (upper panel), Rubisco (lower panel) as control. 1-5, Multiple exposure images. (B) corresponds to Fig 5I. IRT1 protein level under Fe-deficient stress. Col and *mnb1* lines grown vertically on MS (+Fe) medium for 10 days and then shifted to MS (+Fe, control) and Fe-deficient (-Fe) for 7 days. Protein extracts from treated seedlings and analyzed by 10% SDS-PAGE and Western blot assay. Anti-IRT1 antibody (upper panel), Rubisco (lower panel) as control.

**Table S1.** Primer sequences used in this study.

| Primer Name Primer Sequences (5’-3’) |
| --- |
| **For cloning and genotyping**  MNB1GFP-qS CGG**GGTACC**ATGTCTCGATTTGCTATCTTAGT  MNB1GFP-qAS CCG**CTCGAG**ATATTTGATGTAAGCAACAGAA  **For RT-qPCR**  ACTIN8-qS TCAGCACTTTCCAGCAGATG  ACTIN8-qAS CTGTGGACAATGCCTGGAC  MNB1-qS AAGATAGTCGGTGTTGAGCATT  MNB1-qAS AAGTGTACCAAGCAGAGGAGC  FIT-qS TCGGTCTAGGACTTTGATCTCTG  FIT- qAS TCTTGAACATACAACACTGCATCT  IRT1-qS CTCTTTGCTTCCATCAAATGTTC  IRT1- qAS CCTAACGCTATTCCGAATGG  FRO2-qS TTCACCGTTCATGGTCTTTGTT  FRO2- qAS GAGCTATCTCTCCGGCCAAATT  ZIF1-qS GCTGTAAGGTGGAGCAGATGAA  ZIF1-qAS TAGTAGAGGAAGGGATAGAGTGAGG  FRD3-qS CGTCTAGGGATCATCGGTGCA  FRD3-qAS TCCCCGAAGTTTGGTGGAATC  NAS4-qS TGTTCTTGGCTGCTCTTGTAGG  NAS4- qAS CAAGGCTCAACGATTGGATAGA  PYE-qS CAGGACTTCCCATTTTCCAAG  PYE- qAS CTTGTGTCTGGGGATCAGGTT  MYB72-qS GGATAAACTATCTGAGACCGGACG  MYB72- qAS GAGATGCGTGTTCCACACGTTT |
